# Geometric Analysis of Signals for Inference of Multiple Faults in Induction Motors

**DOI:** 10.3390/s22072622

**Published:** 2022-03-29

**Authors:** Jose L. Contreras-Hernandez, Dora L. Almanza-Ojeda, Sergio Ledesma, Arturo Garcia-Perez, Rogelio Castro-Sanchez, Miguel A. Gomez-Martinez, Mario A. Ibarra-Manzano

**Affiliations:** 1 Department of Electronics Engineering, Universidad de Guanajuato, Salamanca 36885, Mexico; jose.contreras@ugto.mx (J.L.C.-H.); dora.almanza@ugto.mx (D.L.A.-O.); selo@ugto.mx (S.L.); arturo@ugto.mx (A.G.-P.); castro@ugto.mx (R.C.-S.); 2 Department of Electrical Engineering, Universidad de Guanajuato, Salamanca 36885, Mexico; gomezma@ugto.mx

**Keywords:** quaternion signal analysis, machine learning comparison, motor fault detection, induction motors

## Abstract

Multiple fault identification in induction motors is essential in industrial processes due to the high costs that unexpected failures can cause. In real cases, the motor could present multiple faults, influencing systems that classify isolated failures. This paper presents a novel methodology for detecting multiple motor faults based on quaternion signal analysis (QSA). This method couples the measured signals from the motor current and the triaxial accelerometer mounted on the induction motor chassis to the quaternion coefficients. The QSA calculates the quaternion rotation and applies statistics such as mean, variance, kurtosis, skewness, standard deviation, root mean square, and shape factor to obtain their features. After that, four classification algorithms are applied to predict motor states. The results of the QSA method are validated for ten classes: four single classes (healthy condition, unbalanced pulley, bearing fault, and half-broken bar) and six combined classes. The proposed method achieves high accuracy and performance compared to similar works in the state of the art.

## 1. Introduction

Induction motors are the most used electromechanical elements in the industry. Early fault detection allows repairing and maintaining the line production with a low cost and high reliability [1]. Mechanical and electrical stresses produce typical defects in induction motors such as broken rotor bars, bearing faults, and rotor unbalance [2]. Fault detection has become an important research topic due to the time and economic cost it saves in critical industrial procedures.

Different methods have been developed for multiple faults detection through signal monitoring such as vibration, current, temperature, voltage, power, and acoustic [3]. The electric current has become the primary signal analyzed by researchers because many faults affect the electromagnetic field, which induces changes in the stator current. Likewise, vibration signals exhibit changes when a failure occurs because of the produced vibration forces. Additionally, the methods for data acquisition of electric current and vibration are noninvasive and are easy to perform. The use of both of these signals improves the detection of multiple faults when the signals or their properties are analyzed using artificial intelligence methods [2].

In the literature, the most popular methods are based on an analysis in the frequency domain. Among these is the work presented in [4], where statistical calculations are applied to data obtained from the application of the Fast Fourier Transform (FFT) to detect seven faults, in which two of them are classified into two levels. The efficiency of this work ranges from 80% to 90%, classified by the support vector machine (SVM). FFT and independent component analysis (ICA) methods are fused to detect a bearing fault, half, one, and two broken rotor bars by current signals [5]. On the other hand, wavelet packet decomposition is applied in [6] to extract coefficients and thus detect three failure modes. SVM is used to classify failures, with 90% of assertiveness.

The authors in [7] use vibration and current signals to obtain fifteen and fourteen statistical features in the time and frequency domain, respectively. Signals in the frequency domain are obtained through the FFT technique. Then, the principal component analysis (PCA) and the linear discriminant analysis (LDA) are applied to reduce features dimension. Finally, an NN-based classifier is used to detect healthy induction motor and bearing faults from 1 mm to 5 mm with a class ratio greater than 98.7%. Similarly, in [8], eleven statistical features are extracted from electric current and vibration signals to diagnose six different faults with an ANN classifier. This method is developed in the time domain, asserting 87.925% to 92.95%. In [9], a convolutional neural network (CNN) is used to detect three motor faults through a technique based on pattern recognition from an electric current signal, a speed measurement, and a vibration signal, achieving an efficiency from 98.8% to 100%.

Machine learning techniques are also applied to motor fault detection, as is shown in [10,11,12]. The authors in [10] developed a method using twenty-one statistical calculations in time and frequency domain obtained from vibration signals with five thousand samples. SVM, K-nearest neighbors (KNN), decision tree, and LDA are proven to detect healthy or broken rotor bars and bearing faults with accuracy from 88.2% to 98.2%. In addition to the techniques mentioned, the fuzzy Artmap network (FAM) is another machine learning method shown in [11], which presents 74.05% of assertiveness. Variations in existing methods are developed as in [12], where sparse deep stacking networks (SDSN) are valued with vibration signals to detect five faults; the obtained results have effectiveness from 93.8% to 100%.

Most research works present the detection of multiple isolated faults because the identification of multiple combined failures implies the existence of conflicts among two or more characteristic values, which is complex to classify [13]. In real rotary machines, multiple combined faults can occur due to the efforts to which they are exposed [14]. There are several works where the identification of multiple combined faults are presented, such as in [15] where two single faults and three combined are classified with more than 99% of accuracy using maximal overlap discrete wavelet transform (MODWT) in current signal and a CNN architecture to classify. The work presented in [16] classifies healthy bearing, healthy rotor, and four different fault combinations with an accuracy of 99.70% using CNN based on adaptive gradient applied to vibration signals. In [13], three combined faults are detected through the entropy analysis of one phase current. A fuzzy algorithm is applied to classify entropy calculus with an assertiveness from 80% to 100%.

The combination of two and three faults presents difficulty in identification because the characteristics of the faults are altered when they are joined. Therefore, few works present this type of classification. In addition, one and two broken rotor bars are complex to identify combined with other faults due to their similar characteristics. The contribution of this research work is a novel methodology based on quaternion rotation and statistical analysis to detect 10 motor faults, among which are multiple failures of broken bars. The electric current and the three-axis vibration signals of an induction motor are analyzed to detect four single classes: (1) healthy condition (HT), (2) unbalanced pulley (BA), (3) bearing fault (BN), and (4) half-broken bar (HB). In addition, the combinations of single motor fault classes generate six additional categories: (5) BN–BA, (6) HB–BA, (7) one broken bar–bearing fault (OB–BN), (8) two broken bar–bearing fault (TB–BN), (9) OB–BN–BA, and (10) TB–BN–BA.

The paper is organized into four sections, as follows: an introduction to fault detection in the motor is given in Section 1; Section 2 describes the theoretical background of the quaternion signal analysis and statistical measure methodology, and how it was adapted to detecting multiple faults in the motor using current and vibration. The results are presented and discussed in Section 3; we compare our results with other common machine learning techniques used for motor fault classification at the end of this section. Likewise, results are correlated with works that present multiple faults. Section 4 draws some conclusions and future works.

## 2. Materials and Methods

**Figure 1:**
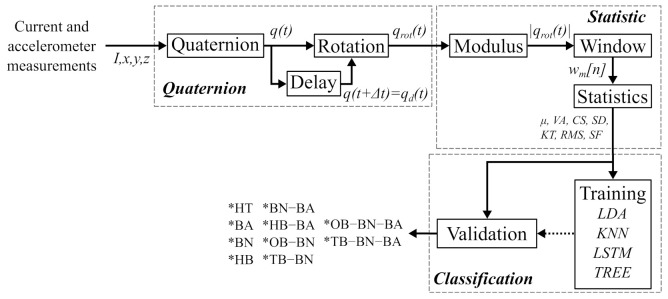
Flowchart of the proposed methodology, where current and acceleration signals are gotten into and single and combined classes are obtained.

The flowchart of the method proposed in this work is shown in Figure 1; this flowchart is divided into two parts: the QSA method formed by quaternion and statistical calculus and the classification method.

### 2.1. QSA Method

Initially, the quaternion q is formed as is shown in (1), where $q_{0}$, $q_{1}$, $q_{2}$, and $q_{3}$ are the coefficients of complex numbers with four components (1, *i*, *j*, and *k*).

(1) $$q=q_{0}+q_{1}i+q_{2}j+q_{3}k$$

The induction motor current *I*(*t*) and the vibration measurements *x*(*t*), *y*(*t*), and *z*(*t*) are discrete signals with m samples which are adapted to the quaternion, as in (2).

(2) q(t)=I(t)+x(t)i+y(t)j+z(t)k

Similarly, the quaternion is delayed Δ*t* samples to obtain a displaced quaternion $q_{d}\left(t\right)$, as in (3).

(3) $$q_{d}\left(t\right)=q\left(t+\Delta t\right)$$

Present quaternion q(t) and rotation $q_{rot}\left(t\right)$ describe the conduct to obtain time-displaced quaternion $q_{d}\left(t\right)$, as is presented in Equation (4).

(4) $$q_{d}\left(t\right)=q_{rot}\left(t\right)⊗q\left(t\right)⊗q_{rot}^{-1}\left(t\right).$$

In the same way, the equation can be presented as is shown in (4).

(5) $$q_{d}\left(t\right)=q_{rot}\left(t\right)\cdot q\left(t\right).$$

Thus, the equation can be expressed as is shown in (6) to obtain a three-dimensional model of orientations and rotations $q_{rot}$, which describes the conduct of present quaternion to obtain delayed quaternion [17].

(6) $$q_{rot}=\left[\begin{array}{ccc} 1-2q_{d2}^{2}-2q_{d3}^{2} & 2(q_{d1}q_{d2}+q_{d0}q_{d3}) & 2(q_{d1}q_{d3}-q_{d0}q_{d2})\\ 2(q_{d1}q_{d2}-q_{d0}q_{d3}) & 1-2q_{d1}^{2}-2q_{d3}^{2} & 2(q_{d2}q_{d3}+q_{d0}q_{d1})\\ 2(q_{d1}q_{d3}+q_{d0}q_{d2}) & 2(q_{d2}q_{d3}-q_{d0}q_{d1}) & 1-2q_{d1}^{2}-2q_{d2}^{2} \end{array}\right]\left[\begin{array}{c} q_{1}\\ q_{2}\\ q_{3} \end{array}\right]$$

Each rotation model is described as is shown in (7).

(7) $$q_{rot}\left(t\right)=q_{r1}\left(t\right)i+q_{r2}\left(t\right)j+q_{r3}\left(t\right)k$$

The statistical analysis of the model describes its behavior, whereby *M* modulus of $q_{rot}\left(t\right)$ are calculated to generate a window $w_{m}$, as is shown in (8).

(8) $$w_{m}\left[n\right]=\left[|q_{rot}\left(t\right)|,|q_{rot}\left(t+\Delta t\right)|,|q_{rot}\left(t+2\Delta t\right)|\ldots |q_{rot}\left(t+M\Delta t\right)|\right]$$

Statistical values such as mean (μ), variance (VA), cluster shape (CP), standard deviation (SD), kurtosis (KT), root mean square (RMS), and shape factor (SF) are calculated from $w_{m}$. Equations (9)–() show the statistical features, and they determine the evolution of signals in the time–space domain [2,8].

(9) $$\begin{array}{cc} & \mu =\frac{1}{N}\sum w_{m} \end{array}$$

(10) $$\begin{array}{cc} & VA=\frac{1}{N}\sum {\left(w_{m}-\mu \right)}^{2} \end{array}$$

(11) $$\begin{array}{cc} & CS=\frac{1}{N}\sum {\left(w_{m}-\mu \right)}^{3} \end{array}$$

(12) $$\begin{array}{cc} & SD=\sqrt{VA} \end{array}$$

(13) $$\begin{array}{cc} & KT=\frac{\sum {\left(w_{m}-\mu \right)}^{4}}{SD^{4}} \end{array}$$

(14) $$\begin{array}{cc} & RMS=\sqrt{\frac{1}{N}\sum {\left(w_{m}\right)}^{2}} \end{array}$$

(15) $$\begin{array}{cc} & SF=\frac{RMS}{\frac{1}{N}\sum \left|w_{m}\right|} \end{array}$$

These statistical evaluations are selected from multiple statistical calculations due to better property separation between faults.

### 2.2. Classification

Finally, some classification algorithms are applied to the statistical features to detect ten motor conditions proposed in this work. Some tools in machine learning, such as a decision tree classifier, KNN, LDA, and LSTM, are used to compare our method efficiency with other classifiers.

The decision tree classifier is a simple classification method that evaluates the feature space with criteria to generate recursive partitions. The evaluation criteria in each internal node are selected by the training set from the top node known as “the root node” to the last nodes called “leaves”. Once the decision tree classifier is trained, new input values are compared to the evaluation criterion in each node to select the most appropriate branch until a leaf node is selected. The last node contains a class label assigned to the input values classification. Leaves nodes can be eliminated to present the best classification results; this method is known as the “pruning decision tree” [18].

The KNN classifier is based on a nonparametric method used in regression tasks. In this classifier, neighborhoods are determined by the distance among elements of the training data assigned to a label. A new instance is classified by selecting the “k closest neighbors” in the training set. Lastly, the most predominant label is attached [19].

The LDA classifier is based on a classical statistical method that minimizes the within-class distance and simultaneously minimizes the between-class distance, discriminating the least amount of information. This method obtains the best linear combination of features that classify a database with multiple classes [20].

The LSTM classifier is an extended architecture of a recurrent neural network (RNN), where the nodes of an RNN are replaced with memory blocks, which contain temporal state in the memory cell and control the information flows in the block with special adaptive multiplicative units called gates. The memory blocks present the input gate to determine the information amount in the cell state, the forget gate to establish which information is deleted, and the output gate to decide the information in the output [21].

### 2.3. Experimental Setup

**Figure 2:**
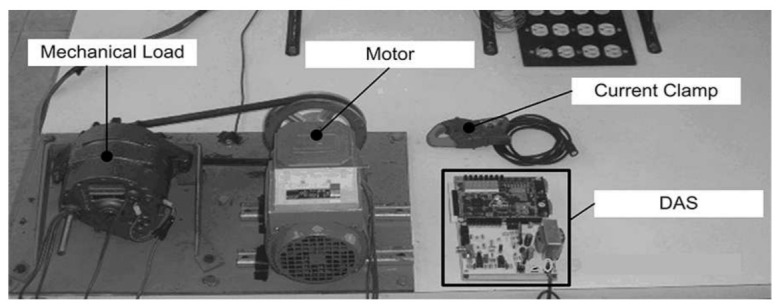
Experimental setup.

Our approach identifies ten motor states through four signals measured from a three-phase induction motor (WEG 00136APE48T) with a weight of 9 kg, a width of 150 mm, and a length of 239 mm. The mentioned motor presents a power of 0.75 hp, with twenty-eight rotor bars and two poles. The signal acquisition of the electric motor current is obtained by a Fluke i200s current clamp measuring the power cable. The three vibration signals are acquired by a three-axis MEMS-based accelerometer (LIS3L02AS4), which is mounted on the induction motor chassis. The four signals are processed from sensors with a 16-bit ADC (ADS7809) and a DAS based on an FPGA system with 4096 samples and a sampling frequency of 1.5 kHz per test during steady-state motor operation running at 3402 rpm. The experimental setup is shown in Figure 2.

**Figure 3:**
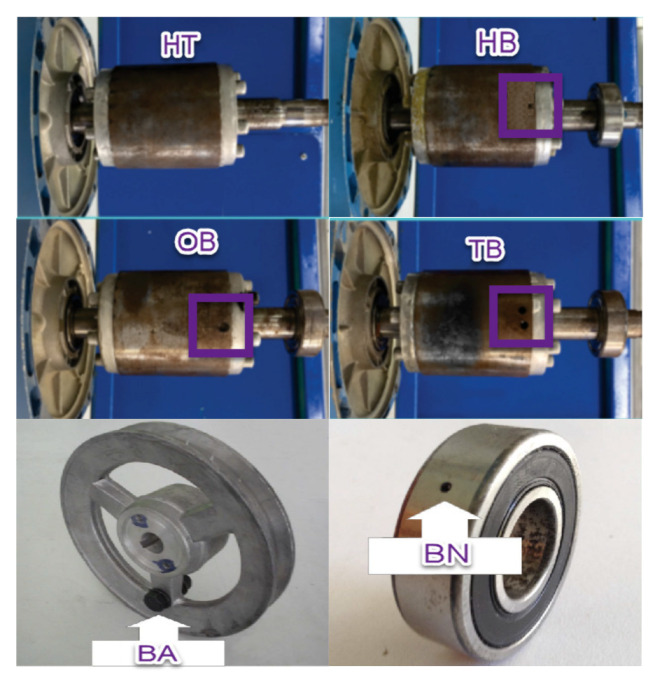
Faults setup. Healthy condition (HT), half broken bar (HB), one broken bar (OB), two broken bar (TB), unbalanced pulley (BA), and bearing fault (BN). Drilled holes in broken bars are shown in purple boxes.

The isolated faults BN, BA, HB, OB, and TB are intentionally provoked, as is shown in Figure 3. BN is produced by a drilled hole with a diameter of 7.938 mm. BA is caused by adding mass to the pulley. HB, OB, and TB are produced by a partially drilled hole into one rotor bar, a total drilled hole into one rotor bar, and total drilled holes into two rotor bars.

**Figure 4:**
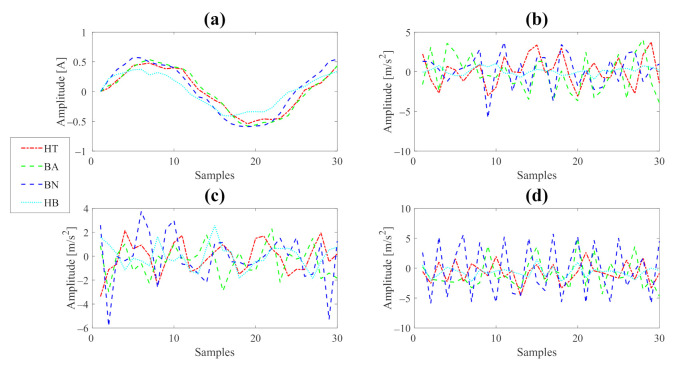
Signals samples. (**a**) Current signals. (**b**) *x*-axis vibration signals. (**c**) *y*-axis vibration signals. (**d**) *z*-axis vibration signals.

The central test bench is structured by one hundred and ten archives, of which HT, BA, HB, and HB–BA each have twenty measurements. On the other hand, BN, BN–BA, OB–BN, TB–BN, OB–BN–BA, and TB–BN–BA have five archives for each. A sample of the entire bench is shown in Figure 4.

## 3. Results

This section presents the results by repeating the proposed method using forty test benches. The number of test files per bench is equalized by the random selection of five archives per class. One archive of each class is randomly selected to train the classifiers, and the remaining four are used for testing. The QSA is applied to the test bench with window samples from one hundred to four thousand.

Table 1 and Table 2 show the results for the accuracy, precision, recall, and F1 of each proved classifications. The results are presented for five different window sizes: one hundred, five hundred, one thousand, two thousand, and four thousand.

The results of QSA applied to the four single classes HT, BA, BN, and HB are presented in Table 1: LDA, KNN, and decision tree classifications with 1.0 accuracy using five hundred window samples. Nevertheless, KNN and decision tree presented a precision, a recall, and an F1 less than those for LDA. These values improve in one thousand window samples. The LSTM classifier presented a high accuracy, but it did not reach the value of 1.

In Table 2, the test bench of the four single classes mentioned above and six combined categories (BN–BA, HB–BA, OB–BN, TB–BN, OB–BN–BA, and TB–BN–BA) is valued. The precision, recall, and F1 of four single classes are shown to analyze the table better. The accuracy in LDA and KNN increase from 0.80 to 0.96, and 0.76 to 0.96, respectively, as the number of samples in the window increases. Both classifiers presented an accuracy of 0.96 in four thousand samples; however, KNN obtains a higher value in precision, recall, and F1. The decision tree gives less efficiency than the LDA and KNN; nevertheless, the accuracy is 0.92 with a window size of one thousand. On the other hand, LSTM needs many data to train, and the effective accuracy is 0.76.

**Figure 5:**
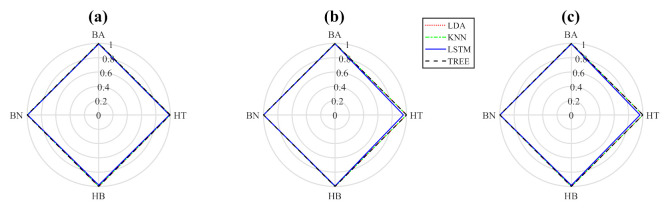
The resulting graphs of four single classes: (**a**) precision, (**b**) recall, and (**c**) F1.

**Figure 6:**
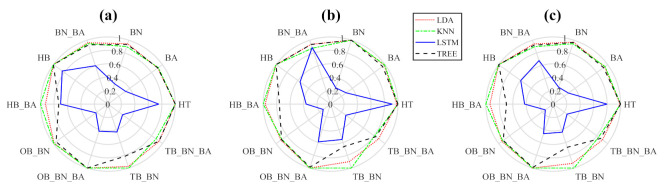
Resulting graph of four single classes and six combinations: (**a**) precision, (**b**) recall, and (**c**) F1.

Figure 5 and Figure 6 show the previously analyzed results using polar graphs where the correct classes are displayed around the graph, where the valued classes are shown around the graphics, and levels present their percentages. In Figure 5, precision, recall, and F1 results are shown when the QSA is applied with four thousand window samples to four individual classes. The method is evaluated with the classifiers mentioned above. The results in graphs show that the four classifiers are optimal to obtain high-precision testing for four individual classes with low variations of recall and F1, as is described in the analysis of Table 1.

In the same way, Figure 6 presents the QSA method applied to four single classes and six combined classes with four thousand window samples using the same classifiers. In this case, the results of the QSA method obtained from LDA and KNN show similar precision in individual and combined faults, while LSTM presents low precision as recall and F1. The decision tree classifier has low precision in two combined faults. Therefore, it is a good option when applying the other faults.

Table 3 shows the comparison of the QSA results presented. The table includes the applied method and its classification. Parentheses separate the number of single and combined classes, and the total number of classes results from the sum of both values. In addition, the accuracy of the method is presented. The neural-network-based classifiers tend to perform well in the attributes classification, using sophisticated and straightforward methods. KNN is the method that presents the best results in this work, classifying four single classes and ten multiple classes (four single and six combined). In the analyzed works, the total amounts of classes range from four to eight with high accuracy values from 0.73 to 1.0. Most of the presented papers use high samples amount; our approach is evaluated with four single classes and five hundred samples, which presents 1.0 accuracy and a range of recall and specificity from 0.995 to 1.0 (0.995–1.0 recall and 0.997–1.0 specificity). The QSA evaluation with combined class shows a 0.96 accuracy in four thousand samples, and recall and specificity range from 0.81 to 1.0 (0.81–1.0 recall and 0.84–1.0 specificity).

## 4. Conclusions

The QSA is a simple method because the signals are processed in the time domain without any space transformation. This method has been proved to isolate faults in an induction motor using a window with few samples, and the results presented high accuracy with narrow ranges of variation. Besides, the QSA was applied to multiple failures, among which are signals of single and combined faults with a total of ten classes to classify. The analyzed signals presented a high accuracy when the appropriate classifier was used. Although the accuracy was not perfect, it remained among the best values that other works gave. In addition, our work is limited in the number of combined failures due to statistical values. A more robust classification algorithm with more statistics could present better results and increase the number of classifications in the combined failures.

The results from the computer simulations clearly show that the QSA is a powerful method to detect isolated and combined faults in induction motors. Our approach presents high accuracy and precision using a window with only a few samples, resulting in short processing time. Because of its characteristics, our approach could be implemented in portable systems and mounted on induction motors of the actual manufacturing process to detect early single and multiple faults without stopping the process involved. As future work, we are interested in increasing the statistics amount and improving the classification method to classify more combined faults supported by an algorithm to select the best features. On the other hand, we want to use the QSA method and regression models to estimate the degree of bar faults.

## Figures and Tables

**Table 1:** Accuracy, precision, recall, and F1 of four single classes.

Clasificator	Samples	Accuracy	Precision				Recall				F1			
			HT	BA	BN	HB	HT	BA	BN	HB	HT	BA	BN	HB
	100	0.99	0.98 ± 0.01	1.00 ± 0.00	1.00 ± 0.00	0.99 ± 0.01	0.99 ± 0.01	1.00 ± 0.00	1.00 ± 0.00	0.98 ± 0.02	0.99 ± 0.01	1.00 ± 0.00	1.00 ± 0.00	0.99 ± 0.01
	500	1.00	1.00 ± 0.01	1.00 ± 0.00	1.00 ± 0.00	1.00 ± 0.01	1.00 ± 0.01	1.00 ± 0.00	1.00 ± 0.00	1.00 ± 0.02	1.00 ± 0.01	1.00 ± 0.00	1.00 ± 0.00	1.00 ± 0.01
LDA	1000	1.00	1.00 ± 0.01	1.00 ± 0.00	1.00 ± 0.00	1.00 ± 0.01	1.00 ± 0.01	1.00 ± 0.00	1.00 ± 0.00	1.00 ± 0.02	1.00 ± 0.01	1.00 ± 0.00	1.00 ± 0.00	1.00 ± 0.01
	2000	1.00	1.00 ± 0.01	1.00 ± 0.00	1.00 ± 0.00	1.00 ± 0.01	1.00 ± 0.01	1.00 ± 0.00	1.00 ± 0.00	1.00 ± 0.02	1.00 ± 0.01	1.00 ± 0.00	1.00 ± 0.00	1.00 ± 0.01
	4000	1.00	1.00 ± 0.01	1.00 ± 0.00	1.00 ± 0.00	1.00 ± 0.01	1.00 ± 0.01	1.00 ± 0.00	1.00 ± 0.00	1.00 ± 0.02	1.00 ± 0.01	1.00 ± 0.00	1.00 ± 0.00	1.00 ± 0.01
	100	0.99	0.99 ± 0.01	1.00 ± 0.00	1.00 ± 0.00	0.97 ± 0.01	0.97 ± 0.01	1.00 ± 0.00	1.00 ± 0.00	0.99 ± 0.02	0.98 ± 0.01	1.00 ± 0.00	1.00 ± 0.00	0.98 ± 0.01
	500	1.00	1.00 ± 0.01	1.00 ± 0.00	1.00 ± 0.00	0.99 ± 0.01	0.99 ± 0.01	1.00 ± 0.00	1.00 ± 0.00	1.00 ± 0.02	1.00 ± 0.01	1.00 ± 0.00	1.00 ± 0.00	1.00 ± 0.01
KNN	1000	1.00	1.00 ± 0.01	1.00 ± 0.00	1.00 ± 0.00	1.00 ± 0.01	1.00 ± 0.01	1.00 ± 0.00	1.00 ± 0.00	1.00 ± 0.02	1.00 ± 0.01	1.00 ± 0.00	1.00 ± 0.00	1.00 ± 0.01
	2000	1.00	1.00 ± 0.01	1.00 ± 0.00	1.00 ± 0.00	1.00 ± 0.01	1.00 ± 0.01	1.00 ± 0.00	1.00 ± 0.00	1.00 ± 0.02	1.00 ± 0.01	1.00 ± 0.00	1.00 ± 0.00	1.00 ± 0.01
	4000	1.00	1.00 ± 0.01	1.00 ± 0.00	1.00 ± 0.00	1.00 ± 0.01	1.00 ± 0.01	1.00 ± 0.00	1.00 ± 0.00	1.00 ± 0.02	1.00 ± 0.01	1.00 ± 0.00	1.00 ± 0.00	1.00 ± 0.01
	100	0.91	0.97 ± 0.01	0.98 ± 0.00	0.98 ± 0.00	0.80 ± 0.01	0.71 ± 0.01	0.96 ± 0.00	0.99 ± 0.00	0.99 ± 0.02	0.79 ± 0.01	0.97 ± 0.00	0.98 ± 0.00	0.88 ± 0.01
	500	0.95	0.97 ± 0.01	1.00 ± 0.00	0.99 ± 0.00	0.88 ± 0.01	0.82 ± 0.01	0.99 ± 0.00	1.00 ± 0.00	1.00 ± 0.02	0.88 ± 0.01	0.99 ± 0.00	1.00 ± 0.00	0.93 ± 0.01
LSTM	1000	0.97	0.97 ± 0.01	1.00 ± 0.00	1.00 ± 0.00	0.93 ± 0.01	0.90 ± 0.01	1.00 ± 0.00	1.00 ± 0.00	1.00 ± 0.02	0.92 ± 0.01	1.00 ± 0.00	1.00 ± 0.00	0.96 ± 0.01
	2000	0.98	1.00 ± 0.01	1.00 ± 0.00	1.00 ± 0.00	0.94 ± 0.01	0.91 ± 0.01	1.00 ± 0.00	1.00 ± 0.00	1.00 ± 0.02	0.93 ± 0.01	1.00 ± 0.00	1.00 ± 0.00	0.96 ± 0.01
	4000	0.99	0.99 ± 0.01	0.99 ± 0.00	0.99 ± 0.00	0.98 ± 0.01	0.96 ± 0.01	0.99 ± 0.00	1.00 ± 0.00	0.99 ± 0.02	0.96 ± 0.01	0.99 ± 0.00	1.00 ± 0.00	0.98 ± 0.01
	100	0.99	0.99 ± 0.01	1.00 ± 0.00	1.00 ± 0.00	0.97 ± 0.01	0.96 ± 0.01	1.00 ± 0.00	1.00 ± 0.00	0.99 ± 0.02	0.98 ± 0.01	1.00 ± 0.00	1.00 ± 0.00	0.98 ± 0.01
	500	1.00	1.00 ± 0.01	1.00 ± 0.00	1.00 ± 0.00	0.99 ± 0.01	0.99 ± 0.01	1.00 ± 0.00	1.00 ± 0.00	1.00 ± 0.02	1.00 ± 0.01	1.00 ± 0.00	1.00 ± 0.00	1.00 ± 0.01
TREE	1000	1.00	1.00 ± 0.01	1.00 ± 0.00	1.00 ± 0.00	1.00 ± 0.01	1.00 ± 0.01	1.00 ± 0.00	1.00 ± 0.00	1.00 ± 0.02	1.00 ± 0.01	1.00 ± 0.00	1.00 ± 0.00	1.00 ± 0.01
	2000	1.00	1.00 ± 0.01	1.00 ± 0.00	1.00 ± 0.00	1.00 ± 0.01	1.00 ± 0.01	1.00 ± 0.00	1.00 ± 0.00	1.00 ± 0.02	1.00 ± 0.01	1.00 ± 0.00	1.00 ± 0.00	1.00 ± 0.01
	4000	1.00	1.00 ± 0.01	1.00 ± 0.00	1.00 ± 0.00	1.00 ± 0.01	1.00 ± 0.01	1.00 ± 0.00	1.00 ± 0.00	1.00 ± 0.02	1.00 ± 0.01	1.00 ± 0.00	1.00 ± 0.00	1.00 ± 0.01

**Table 2:** Accuracy, precision, recall, and F1 of four single classes and six combinations.

Clasificator	Samples	Accuracy	Precision				Recall				F1			
			HT	BA	BN	HB	HT	BA	BN	HB	HT	BA	BN	HB
	100	0.80	0.88 ± 0.05	0.63 ± 0.06	0.90 ± 0.07	0.99 ± 0.01	0.93 ± 0.06	0.69 ± 0.09	0.91 ± 0.08	0.98 ± 0.02	0.90 ± 0.03	0.65 ± 0.04	0.90 ± 0.03	0.99 ± 0.01
	500	0.91	0.99 ± 0.05	0.82 ± 0.06	0.92 ± 0.07	1.00 ± 0.01	0.97 ± 0.06	0.92 ± 0.09	0.99 ± 0.08	1.00 ± 0.02	0.98 ± 0.03	0.86 ± 0.04	0.95 ± 0.03	1.00 ± 0.01
LDA	1000	0.93	1.00 ± 0.05	0.83 ± 0.06	0.93 ± 0.07	1.00 ± 0.01	0.98 ± 0.06	0.93 ± 0.09	0.99 ± 0.08	1.00 ± 0.02	0.99 ± 0.03	0.87 ± 0.04	0.96 ± 0.03	1.00 ± 0.01
	2000	0.94	1.00 ± 0.05	0.85 ± 0.06	0.94 ± 0.07	1.00 ± 0.01	0.97 ± 0.06	0.93 ± 0.09	1.00 ± 0.08	1.00 ± 0.02	0.98 ± 0.03	0.87 ± 0.04	0.97 ± 0.03	1.00 ± 0.01
	4000	0.96	1.00 ± 0.05	0.91 ± 0.06	0.94 ± 0.07	1.00 ± 0.01	0.98 ± 0.06	0.99 ± 0.09	1.00 ± 0.08	1.00 ± 0.02	0.99 ± 0.03	0.94 ± 0.04	0.97 ± 0.03	1.00 ± 0.01
	100	0.76	0.89 ± 0.05	0.56 ± 0.06	0.88 ± 0.07	0.97 ± 0.01	0.81 ± 0.06	0.60 ± 0.09	0.93 ± 0.08	0.98 ± 0.02	0.85 ± 0.03	0.58 ± 0.04	0.90 ± 0.03	0.98 ± 0.01
	500	0.91	0.99 ± 0.05	0.84 ± 0.06	0.92 ± 0.07	1.00 ± 0.01	0.97 ± 0.06	0.92 ± 0.09	0.99 ± 0.08	1.00 ± 0.02	0.98 ± 0.03	0.87 ± 0.04	0.95 ± 0.03	1.00 ± 0.01
KNN	1000	0.94	1.00 ± 0.05	0.86 ± 0.06	0.92 ± 0.07	1.00 ± 0.01	0.99 ± 0.06	0.97 ± 0.09	0.99 ± 0.08	1.00 ± 0.02	0.99 ± 0.03	0.91 ± 0.04	0.95 ± 0.03	1.00 ± 0.01
	2000	0.95	1.00 ± 0.05	0.89 ± 0.06	0.92 ± 0.07	1.00 ± 0.01	1.00 ± 0.06	0.97 ± 0.09	1.00 ± 0.08	1.00 ± 0.02	1.00 ± 0.03	0.92 ± 0.04	0.96 ± 0.03	1.00 ± 0.01
	4000	0.96	1.00 ± 0.05	0.92 ± 0.06	0.90 ± 0.07	1.00 ± 0.01	1.00 ± 0.06	0.99 ± 0.09	1.00 ± 0.08	1.00 ± 0.02	1.00 ± 0.03	0.95 ± 0.04	0.94 ± 0.03	1.00 ± 0.01
	100	0.71	0.87 ± 0.05	0.61 ± 0.06	0.80 ± 0.07	0.95 ± 0.01	0.76 ± 0.06	0.64 ± 0.09	0.86 ± 0.08	0.93 ± 0.02	0.78 ± 0.03	0.59 ± 0.04	0.81 ± 0.03	0.92 ± 0.01
	500	0.76	0.96 ± 0.05	0.62 ± 0.06	0.90 ± 0.07	0.98 ± 0.01	0.83 ± 0.06	0.66 ± 0.09	0.86 ± 0.08	0.98 ± 0.02	0.85 ± 0.03	0.61 ± 0.04	0.85 ± 0.03	0.98 ± 0.01
LSTM	1000	0.75	0.95 ± 0.05	0.64 ± 0.06	0.86 ± 0.07	1.00 ± 0.01	0.91 ± 0.06	0.61 ± 0.09	0.83 ± 0.08	0.94 ± 0.02	0.92 ± 0.03	0.57 ± 0.04	0.79 ± 0.03	0.96 ± 0.01
	2000	0.73	0.95 ± 0.05	0.55 ± 0.06	0.70 ± 0.07	0.98 ± 0.01	0.87 ± 0.06	0.64 ± 0.09	0.82 ± 0.08	0.96 ± 0.02	0.89 ± 0.03	0.54 ± 0.04	0.72 ± 0.03	0.96 ± 0.01
	4000	0.48	0.76 ± 0.05	0.32 ± 0.06	0.32 ± 0.07	0.84 ± 0.01	0.92 ± 0.06	0.29 ± 0.09	0.25 ± 0.08	0.56 ± 0.02	0.80 ± 0.03	0.28 ± 0.04	0.26 ± 0.03	0.60 ± 0.01
	100	0.76	0.90 ± 0.05	0.57 ± 0.06	0.87 ± 0.07	0.97 ± 0.01	0.79 ± 0.06	0.61 ± 0.09	0.93 ± 0.08	0.99 ± 0.02	0.84 ± 0.03	0.58 ± 0.04	0.89 ± 0.03	0.98 ± 0.01
	500	0.88	0.98 ± 0.05	0.80 ± 0.06	0.86 ± 0.07	1.00 ± 0.01	0.95 ± 0.06	0.89 ± 0.09	0.98 ± 0.08	1.00 ± 0.02	0.96 ± 0.03	0.83 ± 0.04	0.91 ± 0.03	1.00 ± 0.01
TREE	1000	0.92	1.00 ± 0.05	0.84 ± 0.06	0.87 ± 0.07	1.00 ± 0.01	0.99 ± 0.06	0.93 ± 0.09	0.97 ± 0.08	1.00 ± 0.02	1.00 ± 0.03	0.87 ± 0.04	0.91 ± 0.03	1.00 ± 0.01
	2000	0.91	0.99 ± 0.05	0.87 ± 0.06	0.90 ± 0.07	1.00 ± 0.01	0.96 ± 0.06	0.95 ± 0.09	0.99 ± 0.08	1.00 ± 0.02	0.98 ± 0.03	0.90 ± 0.04	0.94 ± 0.03	1.00 ± 0.01
	4000	0.90	1.00 ± 0.05	0.92 ± 0.06	0.93 ± 0.07	1.00 ± 0.01	1.00 ± 0.06	0.95 ± 0.09	1.00 ± 0.08	1.00 ± 0.02	1.00 ± 0.03	0.92 ± 0.04	0.96 ± 0.03	1.00 ± 0.01

**Table 3:** Methods comparison table involving the proposed method.

Method	Classification	Single (Comb)	Samples	Accuracy
Statistical Method [22]	SVM	3 (1)	500	0.85–1.00
MultirowMP and DWT [3]	SVM, KNN and Ensemble	3 (3) 5 (1)	3000	0.97–1.00
Time vibration signal [16]	ADG-dCNN	3 (3)	2100	0.98–0.99
Time and frequency analyses [23]	OAA-MCSVM	3 (4)	1,250,000	0.73–1.00
Homogeneity and kurtosis analysis [24]	ANN	5	11,059	1.00
Frequency and time features, GA-PCA, LDA [25]	NN	4 (4)	375,000−500,000	0.96–0.98
SDAE [26]	NMEC-DNN	4 4 (4)	250–500	0.91–1.0 00.88–0.95
**QSA** **(Our approach)**	**KNN**	**4** **4 (6)**	**500** **4000**	**1.00** **0.96**

## Data Availability

Motor signals database and their corresponding diagnosis were provided in collaboration with national and international partners based on a privacy agreement with the University of Guanajuato. The agreement avoids publicly sharing or distributing any kind of data.

## Abbreviations

The following abbreviations are used in this manuscript:

QSA	Quaternion Signal Analysis
FFT	Fast Fourier Transform
SVM	Support Vector Machine
ICA	Independent Component Analysis
ANN	Artificial Neural Network
CNN	Convolutional Neural Network
MLP	Multilayer Perceptron
KNN	k-Nearest Neighbors
SMO	Sequential Minimal Optimization
FAM	Fuzzy ArtMap Network
SDSN	Sparse Deep Stacking Network
HT	Healthy Condition
BA	Unbalanced Pulley
BN	Bearing Fault
HB	Half Broken Bar
OB	One Broken Bar
TB	Two Broken Bar
μ	Mean
VA	Variance
CS	Cluster Shape
SD	Standard Deviation
KT	Kurtosis
RMS	Root Mean Square
SF	Shape Factor
LDA	Linear Discriminant Analysis
LSTM	Long Short-Term Memory
RNN	Recurrent Neural Network
